# Modeling Injection Molding of High-Density Polyethylene with Crystallization in Open-Source Software

**DOI:** 10.3390/polym13010138

**Published:** 2020-12-31

**Authors:** Kristjan Krebelj, Anton Krebelj, Miroslav Halilovič, Nikolaj Mole

**Affiliations:** 1 Faculty of Mechanical Engineering, University of Ljubljana, Aškerčeva, 1000 Ljubljana, Slovenia; kristjan.krebelj@fs.uni-lj.si (K.K.); miroslav.halilovic@fs.uni-lj.si (M.H.); 2 Tehnoplast, Razvojno raziskovalna skupina, Neverke 30, 6256 Košana, Slovenia; anton.krebelj@tehnoplast.si

**Keywords:** injection molding, numerical simulation, polymer crystallization, open-source code

## Abstract

This work investigates crystallization modeling by modifying an open-source computational fluid dynamics code *OpenFOAM*. The crystallization behavior of high-density polyethylene (HDPE) is implemented according to theoretical and experimental literature. A number of physical interdependencies are included. The cavity is modeled as deformable. The heat transfer coefficient in the thermal contact towards the mold depends on contact pressure. The thermal conductivity is pressure- and crystallinity-dependent. Specific heat depends on temperature and crystallinity. Latent heat is released according to the crystallization progress and temperature. Deviatoric elastic stress is evolved in the solidified material. The prediction of the cavity pressure evolution is used for the assessment of the solution quality because it is experimentally available and governs the residual stress development. Insight into the thermomechanical conditions is provided with through-thickness plots of pressure, temperature and cooling rate at different levels of crystallinity. The code and simulation setup are made openly available to further the research on the topic.

## 1. Introduction

Injection molding simulation research requires a considerably advanced computer code, which is particularly true when including crystallization modeling. Many publications made use of in-house codes that were not made public, while the industrial injection molding solvers specialize for the industrial needs and prove to be restrictive for research of the thermomechanical phenomena in injection molding. The commercial general purpose computational fluid dynamics (CFD) codes may allow customization for conducting an injection molding simulation, but the undertaking is challenging and likewise results an in-house code.

Injection molding simulation has been the subject of ongoing research for decades. Kennedy and Zheng [1] have thoroughly reviewed the history of injection molding simulation publications. Based on the aim of predicting part geometry, the research was focused on the residual stress prediction. An important publication was contributed by Baaijens [2] who developed a model capable of describing the filling and packing stages as well as the final residual stresses. They highlighted the effect of mold compliance on the cavity pressure evolution. Later, the constitutive modeling was advanced with an advanced viscoelastic material model by Chang and Chiou [3], a three-dimensional finite volume method was applied [4] and residual stresses were analyzed both experimentally and numerically for amorphous and crystalline polymers [5]—Guevara-Morales and Figueroa-López [6] published a thorough review of the research on the residual stresses.

An important contribution to injection molding research was made by the development of the *UNISA* code, described by Pantani et al. [7], which scientifically tackled the crystallization specifics in addition to the general problem of injection molding. The code is advanced in terms of physics, but does not appear to be publicly accessible. It was used by De Santis et al. [8] for predicting the shrinkage of injection molded isotactic polypropylene (iPP) and inspecting the role of holding time and pressure. Pantani et al. [9,10] also investigated crystallization modeling in injection molding simulation, compiling a comprehensive review of the subject. The polymer of interest was iPP. Zheng et al. [11] modeled flow induced crystallization while also accounting for the colorant content, likewise investigating iPP. Zheng et al. [12] reviewed the modeling aspect in a dedicated chapter while Janeschitz-Kriegl [13] compiled a thorough introduction to the physics of polymer crystallization and provided material data for different polymers, demonstrating the complexity of the phenomenon form the experimental and numerical perspective.

An application-oriented study of high-density polyethylene (HDPE) injection molding was performed by Kabanemi et al. [14]. The study focused on the part geometry prediction in the scope of solid mechanics, while the fluid thermo-mechanics was of secondary importance. Kamal et al. [5] simulated injection molding of HDPE using a finite volume code McKam4. The software was capable of describing crystallization, but has not been referenced in later studies and does not appear to be publicly available. Ilinca and Hetú [15] simulated gas-assisted injection molding of HDPE where they mostly elaborated on the numerical procedure.

While the studies concerned with crystallization in injection molding simulation focused on iPP, HDPE has received far less attention. The published work tended to employ commercial codes or inhouse codes. On this basis, we formulate two aims of this study:

- Promote research collaboration by sharing an open-source code solution for injection molding crystallization modeling;
- Investigate crystallization modeling of HDPE and the related material data;
- Provide insight into the thermomechanical development.

To overcome the hindrance of commercial code lock-in and aid the research community cooperation, we have customized the open-source general purpose CFD code *OpenFOAM* [16] and made it publicly available as *openInjMoldSim* [17]. The solver is a modification of *compressibleInterFoam* of *OpenFOAM v3.0.1* [16]. Within the scope of our previous work [18], we demonstrated this approach on modeling volumetric relaxation of amorphous polystyrene during injection molding using a modified *openInjMoldSim* code, named *openInjMoldDyMSimAmClr* [19]. The present publication demonstrates a code version for simulating injection molding of HDPE with crystallization, denoted as *openInjMoldDyMSimCr* [20]. This highlights the research potential of an open-source injection molding code that can be tailored to the specific research needs.

The numerical solver is suitable for non-simplified three-dimensional geometry and incorporates three influences on the cavity pressure evolution: mold compliance, pressure-dependent thermal contact with the mold and the crystallization dependent specific volume. This allows comparing the prediction to the experimental pressure evolution.

The course of this work (section: Materials and methods) consists of three major parts. The experimental results are outlined and the reader is referred to the publication with further details. The crystallization model introduces the theoretical background of the HDPE crystallization model. Subsequently, we describe the formulation of a numerical model of injection molding to assess the quality of the prediction of the pressure evolution by comparing it to the experimental data. The results are reviewed and discussed, offering quantitative insight into the thermo-mechanics of an injection molded HDPE product. The paper is concluded with the implications for the research field and directions for future work.

## 2. Materials and Methods

### 2.1. Experimental Investigation

**Figure 1:**
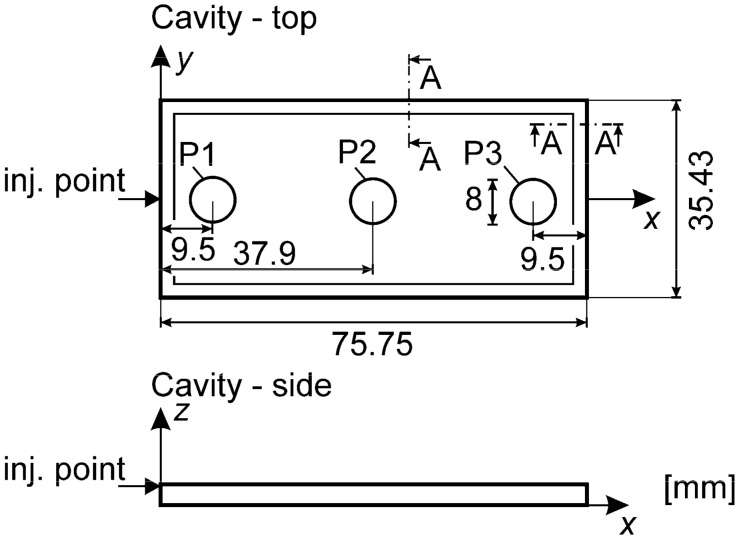
Mold cavity geometry.

The experimental counterpart of the examined case consists of pressure evolutions that were measured and published before [21]. The temperature of the mold cooling water was set to 50 °C, leading to about 52 °C at the surface of the cavity while the melt temperature was set to 220 °C. The crystalline plastic HDPE Sabic M80064 was used. The experimental mold cavity is shown in Figure 1 with the pressure measurement positions denoted by P0, P1, P3 and P4. The pressure measuring method was indirect, i.e., using rods installed in the mold that transmitted the force from the cavity to the sensor. The relevant pressure evolutions are reported together with the numerical results later on.

### 2.2. HDPE Thermal and Volumetric Behavior

Crystallization influences the thermal and mechanical conditions. The transition from melt to solid implies a change in the specific heat, a release of latent heat [22] and a decrease in the specific volume. An increase in thermal conductivity due to solidification has also been measured [23], and is taken into account here.

#### 2.2.1. Crystallization

The crystallinity degree was evolved using the Kolmogofoff-Avrami-Evans model [1] expressed in the form of the Scheinder’s differential equation system [24]. The fictive crystalline volume field *φ* is integrated via intermediate variables $\varphi_{1}$ and $\varphi_{2}$ according to,

(1) $${\dot{\varphi }}_{2}=G_{k}\cdot 8\pi N_{k},\;{\dot{\varphi }}_{1}=G_{k}\varphi_{2},\;\dot{\varphi }=G_{k}\varphi_{1},$$

where $N_{k}$ is the nuclei density and $G_{k}$ the spherulite lineal growth rate. The volume fraction of the solidified material is calculated as:

(2) $$w_{V}=1-\exp\left(-\varphi \right).$$

The mass fraction of the solidified material is taken as the relative crystallinity,

(3) $$\xi =\frac{w_{V}\rho^{\left(s\right)}}{w_{V}\rho^{\left(m\right)}+\left(1-w_{V}\right)\rho^{\left(s\right)}},$$

where $\rho^{\left(m\right)}$ and $\rho^{\left(s\right)}$ are the melt and solid density, respectively. The pressure influence modeling was adopted from Zuidema et al. [25], with a decrease in the effective temperature as,

(4) $$T^{c}=T-b_{6}p,$$

where $b_{6}$ is adopted from the Tait equation while $T^{c}$ is the temperature input to the crystallization model. The nucleation density (nuclei number per volume unit) description was adopted from Koscher and Fulchiron [26],

(5) $$N_{k}\left(T^{c}\right)=N_{0}\exp\left(\frac{T_{m}^{0}-T^{c}}{\Delta T_{0}}\right),$$

where $T_{m}^{0}$ is the HDPE melting temperature at 419 K [27]. The free parameters $N_{0}$ and $\Delta T_{0}$ were identified in the scope of this work by matching the model crystallization rate with the HDPE calorimetry measurements [28,29] (Table 1).

The spherulite lineal growth rate was described according to Mandelkern et al. [30] as reported by Van Krevelen and Nijenhuis [27] to which an additional term was added as suggested by Mandelkern [31],

(6) $$G_{k}\left(T^{c},\xi \right)=G_{k,0}\exp\left(-\frac{E_{D}}{R_{p}T^{c}}-\frac{C_{3}T_{m}^{0}}{T^{c}\left(T_{m}^{0}-T^{c}\right)}-r_{c}\xi \right)$$

with $R_{p}$ as the universal gas constant. The parameters $G_{k,0}$, $C_{3}$ and $E_{D}$, were provided for polyethylene by Van Krevelen and Nijenhuis [27] (Table 1). The parameter $r_{c}$ phenomenologically accounts for the fractions of untransformable material in the kinetics [31] and was identified in this work for HDPE by matching the calorimetry data to the crystallization model output.

**Figure 2:**
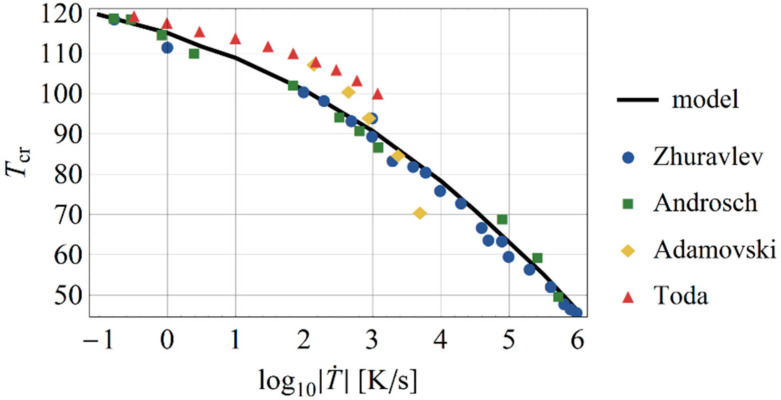
Half-crystallization temperature compared to experimental data.

The temperature at half-crystallization $T_{cr}=T\left(\xi =0.5\right)0$ was plotted against available experimental data for a broader range of cooling rates (Figure 2) and compared to available fast cooling rate measurements on polyethylene [28,29,32,33]. Zhuravlev et al. [29] tested an HDPE with a molecular mass of 500 kDa, while Androsch et al. [33] tested ultra-high molecular weight polyethylene (UHMWPE) and obtained similar half crystallization times. Toda et al. [28] examined a polyethylene of 50 kDa.

#### 2.2.2. Specific Volume

The specific volume is expressed as,

(7) $$v_{s}=v^{\left(s\right)}\xi +v^{\left(m\right)}\left(1-\xi \right)$$

where $v^{\left(s\right)}$ and $v^{\left(m\right)}$ are the solid and melt specific volume, respectively [12,34]. The $v^{\left(s\right)}$ and $v^{\left(m\right)}$ are identifiable from the two-domain Tait equation (see Zheng et al. [12]) which is also derived based on the rule of mixture,

(8) $$v^{\left(m\right)}=\left(b_{1m}+b_{2m}\left(T-b_{5}\right)\right)\left(1-C\ln\left(1+\frac{p}{b_{3m}\exp\left(-b_{4m}\left(T-b_{5}\right)\right)}\right)\right)$$

and

(9) $$v^{\left(s\right)}=\left(b_{1s}+b_{2s}\left(T-b_{5}\right)\right)\left(1-C\ln\left(1+\frac{p}{b_{3s}\exp\left(-b_{4s}\left(T-b_{5}\right)\right)}\right)\right).$$

The subscripts “m” and “s” denote the b parameters’ values for the melt and solid domains, respectively, with the universal constant C=0.0894. The parameters are listed in Table 2 for HDPE Sabic M80064 [35].

**Figure 3:**
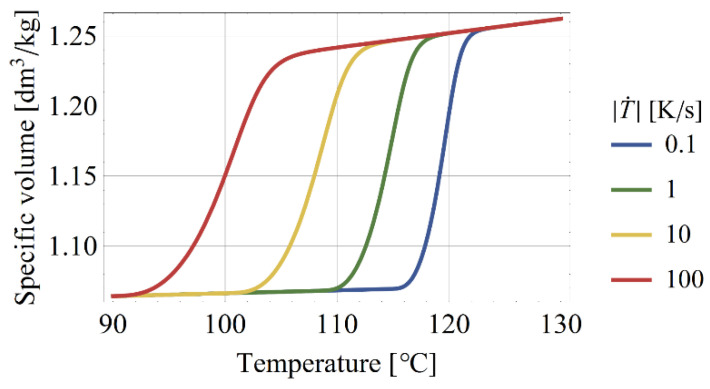
Specific volume at atmospheric pressure for different cooling rates.

Integrating the crystallization degree at constant cooling (Equations (1) and (3)), the specific volume of HDPE (Equation (7)) is plotted against temperature for different cooling rates in Figure 3, revealing the crystallization effect. As the temperature is considered independent, the thermal properties do not affect this result.

#### 2.2.3. Specific Heat

**Figure 4:**
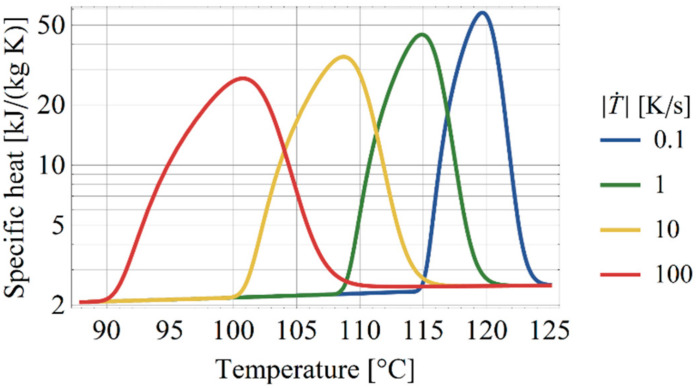
Apparent specific heat at constant cooling with crystallization.

Latent heat is released with crystallization and has a strong influence on the thermal conditions. Gaur and Wunderlich [22], described the apparent specific heat by lumping the latent into the to the mixture equation,

(10) $$c_{p}=c_{p}^{c}w^{c}+c_{p}^{a}\left(w^{c}-1\right)-\frac{dw^{c}}{dT}\Delta H_{f}$$

where $c_{p}$ is the specific heat of the mixture at constant pressure, $c_{p}^{c}$ and $c_{p}^{a}$ are the specific heat at constant pressure for the crystalline, and the amorphous phase, respectively, $w^{c}$ is the mass fraction of the crystalline phase, and $\Delta H_{f}$ is the heat of fusion. In this work, the crystalline mass fraction is calculated according to,

(11) $$w^{c}=w_{\infty }^{c}\xi$$

by assuming the semi-crystalline phase to contain a fraction of $w_{\infty }^{c}=0.66$ of the crystallized material. In the numerical implementation of Equation (10), the first two terms are used to describe the specific heat with the last term transferred to a heat source adopting the values recommended by Gaur and Wunderlich [22]. The resulting apparent specific heat is shown in Figure 4 for different cooling rates.

### 2.3. Injection Molding Model Formulation

The development of crystallization was investigated in a simulation of the injection molding filling and packing stages. This is a problem of a laminar, non-isothermal, compressible flow of two immiscible fluids according to the volume of fluid method. The fluid mechanics problem is based on the conservation equations complemented by the constitutive equations. A system of partial differential equations is obtained and discussed from the geometrical aspect. The code and the numerical setup are publicly available under the name *openInjMoldDyMSimCr* [20] as a further modified version of *openInjMoldSim* [17].

#### 2.3.1. Conservation Equations

The code employs the finite volume method to solve the differential conservation equations for the mass, momentum and energy. The conservation of mass relates the density ρ to the velocity field $u_{k}$,

(12) $$\frac{\partial \rho }{\partial t}+\frac{\partial \left(\rho u_{k}\right)}{\partial x_{k}}=0$$

where Einstein’s notation is used with k=1,2,3 for the Cartesian coordinates $x_{k}$. The conservation of momentum relates the velocity field to the stresses $\sigma_{ij}$,

(13) $$\rho \frac{Du_{i}}{Dt}=\frac{\partial \sigma_{ij}}{\partial x_{j}}$$

where the notation for the material derivative is used:

(14) $$\frac{Du_{i}}{Dt}=\frac{\partial u_{i}}{\partial t}+u_{k}\frac{\partial u_{i}}{\partial x_{k}}.$$

The stresses are composed of the hydrostatic pressure p, the elastic contribution $\tau_{ij}^{e}$ in the solidified material and the viscous contribution $\tau_{ij}^{v}$ as,

(15) $$\sigma_{ij}=-p\;\delta_{ij}+\tau_{ij}^{e}+\tau_{ij}^{v}$$

with the Kronecker delta tensor $\delta_{ij}$. Finally, the energy conservation is imposed with the equation,

(16) $$\rho c_{p}\frac{DT}{Dt}+\frac{T}{\rho }{\left(\frac{\partial \rho }{\partial T}\right)}_{p}\frac{Dp}{Dt}=\tau_{ij}^{v}D_{ij}+\frac{\partial }{\partial x_{i}}\left(k\frac{\partial T}{\partial x_{i}}\right)+\rho \Delta H_{f}\frac{Dw^{c}}{Dt}$$

with the specific heat $c_{p}$, thermal conductivity k and the rate of strain tensor:

(17) $$D_{ij}=\frac{1}{2}\left(\frac{\partial u_{i}}{\partial x_{j}}+\frac{\partial u_{j}}{\partial x_{i}}\right).$$

**Figure 5:**
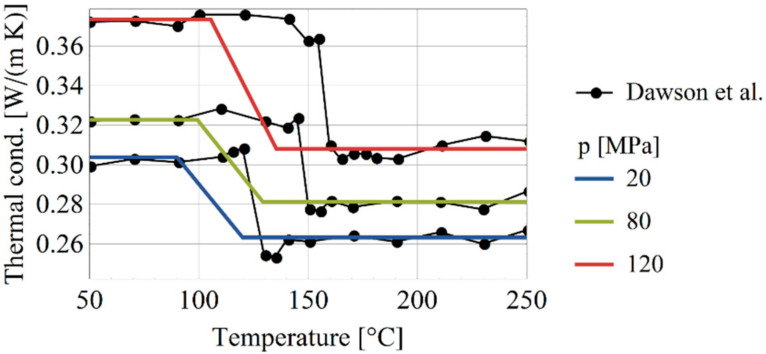
The thermal conductivity as measured [23] and modeled (thick horizontal lines).

The thermal conductivity was adopted from Dawson et al. [23] as shown in Figure 5. The depicted thermal dependence only approximately illustrates the effect of crystallization. Note that Dawson et al. [23] performed the measurements during heating of HDPE which lead to a phase change at higher temperatures than during cooling.

#### 2.3.2. Viscosity

The frequently used Cross-WLF melt viscosity model was employed,

(18) $$\;\eta_{0}\left(T,\;p\right)=D_{1}\exp\left(-\frac{A_{1}\left(T-D_{2}-D_{3}p\right)}{{\tilde{A}}_{2}+T-D_{2}}\right),$$

(19) $$\eta \left(\dot{\gamma },T,\;p\right)=\frac{\eta_{0}\left(T,p\right)}{1+{\left(\frac{\eta_{0}\left(T,p\right)\dot{\gamma }}{\tau^{*}}\right)}^{n-1}}$$

with the model parameters listed in Table 3 as obtained from [35] and corrected using rotational rheometry.

#### 2.3.3. Constitutive Modeling

The viscosity and specific volume were modeled as described in the previous section with the viscous deviatoric stress was calculated as,

(20) $$\tau_{ij}^{v}=2\;\eta \;D_{ij}^{d}$$

where $D_{ij}^{d}$ is the deviatoric component of the rate of deformation tensor. The melt was assumed to solidify when reaching the maximum viscosity of 0.5 MPas, at which point the deviatoric elasticity was onset according to the Upper Convected Maxwell model [36] with an infinite relaxation time,

(21) $$\frac{\partial \tau_{ij}^{e}}{\partial t}+u_{k}\frac{\partial \tau_{ij}^{e}}{\partial x_{k}}-\frac{\partial u_{i}}{\partial x_{k}}\tau_{kj}^{e}-\frac{\partial u_{j}}{\partial x_{k}}\tau_{ik}^{e}=2GD_{ij}^{d}$$

where $\tau_{ij}^{e}$ is the elastic deviatoric stress in Einstein’s notation, $u_{i}$ is the velocity vector, $x_{i}$ are the Cartesian coordinates, G=200 MPa is the shear modulus [37] and $D_{ij}^{d}$ is the deviatoric rate of deformation tensor.

The growing fraction of the crystalline content in the melt gradually leads to flow cessation. The increase in viscosity is a challenging phenomenon to investigate experimentally. The viscosity was scaled due to crystallinity using the empirical factor,

(22) $$\frac{\eta_{c}}{\eta }=1+f\exp\left(-h/w_{V}^{m}\right)$$

as suggested by Titomanlio et al. [38] (Table 4).

#### 2.3.4. Geometry and Boundary Conditions

**Figure 6:**
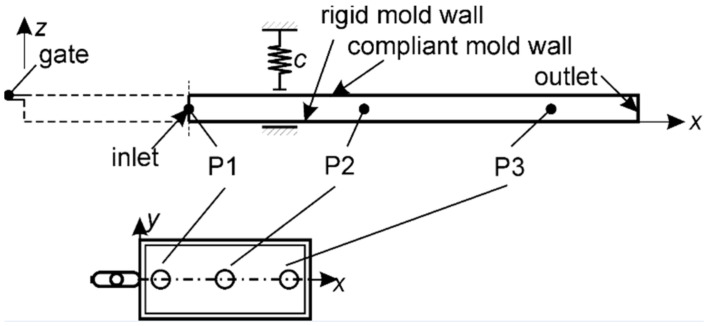
Computational domain.

The cavity was described by the longitudinal cross-section displayed in Figure 6. The measured P1 pressure was used as the boundary condition. This eliminated the gate from the computational domain where the flow is not planar. The air was released through the outlet during the filling stage which was closed during the packing stage.

The melt filling temperature was 220 °C and the mold temperature 50 °C. The heat transfer coefficient h on the mold walls was modeled as pressure dependent with a linear interpolation between the points $\left(p,h\right)=\left(0\;\mathrm{MPa},1020\;W/\left(m^{2}K\right)\right)$ and $\left(100\;\mathrm{MPa},\;5384\;W/\left(m^{2}K\right)\right)$ as identified from the work of Delaunay et al. [39]. The compliance of the cavity was set to c=0.75 μm/MPa, similarly as in [40]. The finite volume mesh consisted of 64 cells in the thickness direction and 2400 cells in the length direction.

## 3. Results and Discussion

**Figure 7:**
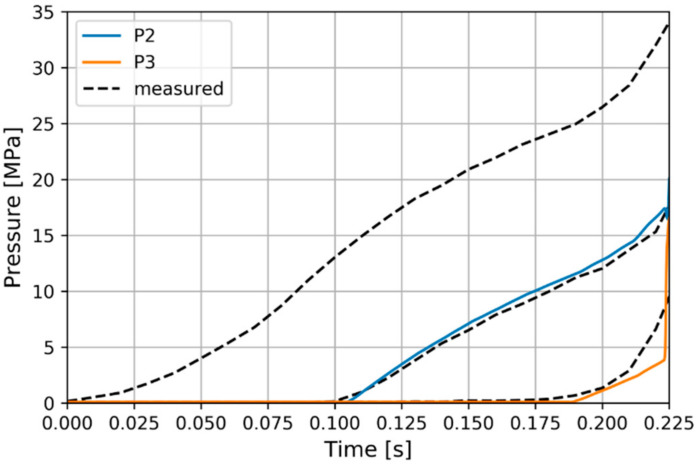
Filling pressure evolutions at the experimental positions.

The calculated pressures at P2 and P3 positions are compared to the measured values (Figure 7). The P1 pressure was imposed at the inlet according to the measurements. This result offers insight into the quality of the pressure gradient and filling rate prediction.

**Figure 8:**
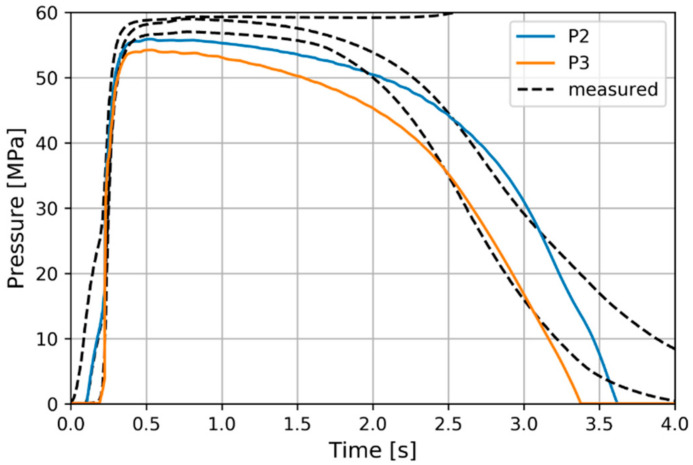
Packing pressure evolutions at the experimental positions.

The evolution of the packing pressure at these positions is depicted in Figure 8. The solidification time appears to have been well matched with the experimental values, while pressure gradient during post-filling was found to be slightly over-predicted. Further investigation of the post-filling phase pressure gradient would involve an experimental and numerical investigation of the *D*_3_ parameter in the Cross-WLF Equation (18) as it increases the high-pressure viscosity.

**Figure 9:**
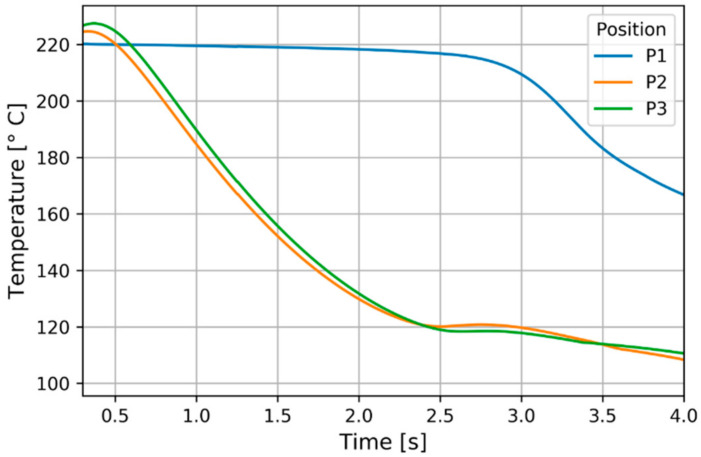
Temperature evolution at the observed positions (mid-thickness).

At the start of the cooling a temperature increase above the initial melt temperature is predicted due to adiabatic compression. The latent heat release was most evident at 120 °C in the mid-thickness as shown in Figure 9 for the experimental pressure positions. A plateau developed as typically in quenching of crystalline materials. A slight temperature increase was predicted at the start of the plateau which could be realistic [41].

**Figure 10:**
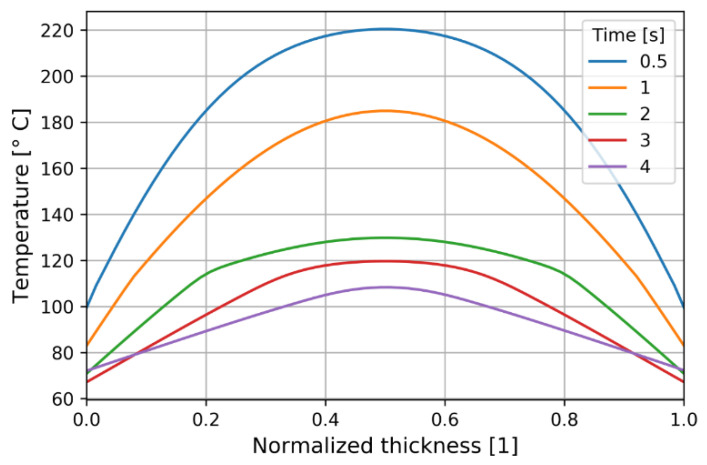
Temperature distribution at P2 through normalized thickness (dimensionless).

A far faster temperature drop was found at the mold contact (Figure 10) where a temperature rise during packing developed as a consequence of the decreasing heat transfer coefficient at dropping pressure (see [39,42]).

**Figure 11:**
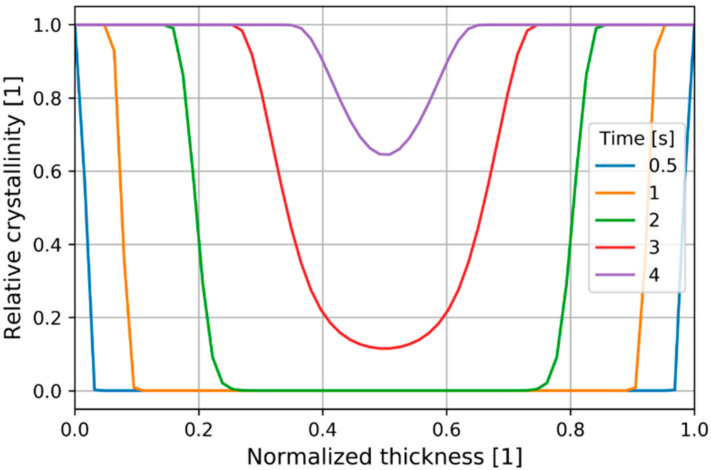
Relative crystallinity distribution at P2.

At 4 s the crystallization on the P2 position was almost complete (Figure 11) and the graphs indicate that the crystallization near the mold surfaces was far more rapid.

**Figure 12:**
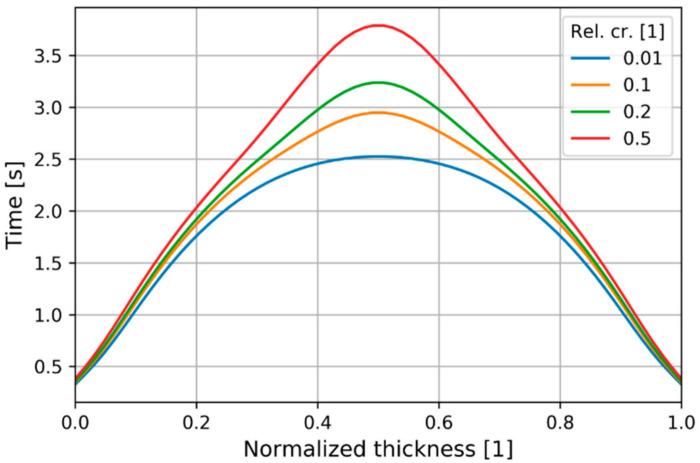
Time to reach different levels of relative crystallinity along the normalized thickness at P2.

The times of reaching different relative crystallinity degrees are depicted in Figure 12, indicating that the center of the cavity took more than a second to advance the relative crystallinity from 1% to 50%, unlike the surface layers with rapid crystallization.

**Figure 13:**
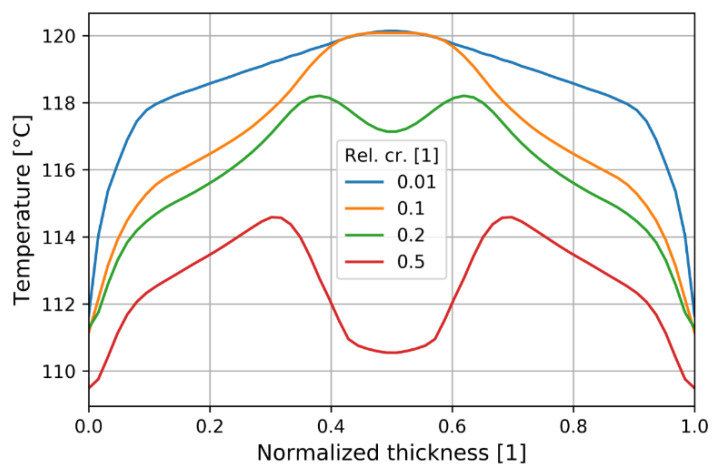
Temperature distribution through normalized thickness depending on relative crystallinity at P2.

Temperature at solidification is an important piece of information when modeling is simplified by assuming the so called “no flow temperature” [43]. Figure 13 depicts the temperature through the thickness when the 50% relative crystallinity was reached. It was found to be confined between 110 °C and 120 °C.

**Figure 14:**
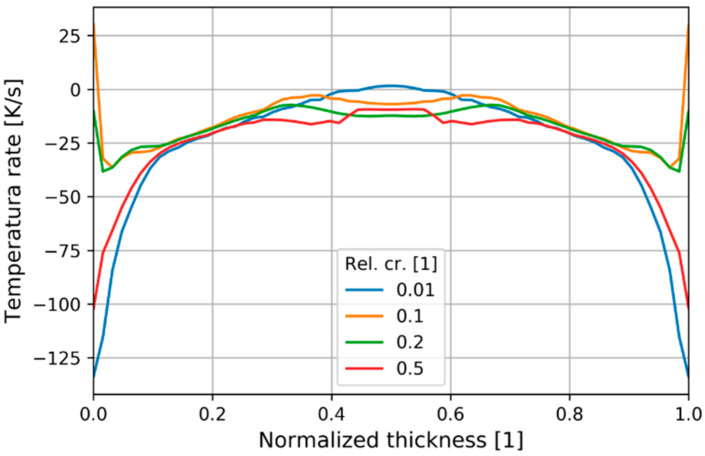
Temperature rate distribution through normalized thickness depending on relative crystallinity at P2.

The time derivative of temperature at different values of relative crystallinity (Figure 14) reveals that the near surface layers were cooling at rates up to −130 K/s, while the latent heat lead to a brief and rapid increase in temperature.

**Figure 15:**
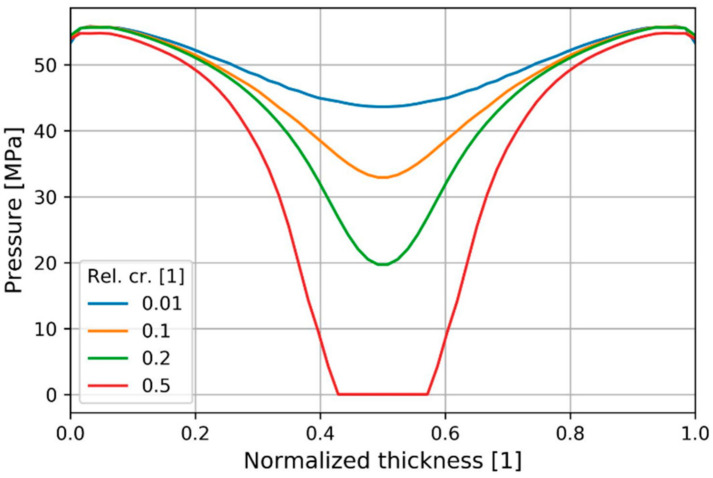
Pressure distribution through normalized thickness depending on relative crystallinity at P2.

The pressure change during crystallization was smaller in the near-mold material (Figure 15). This result provides insight into the impact of assuming instant solidification in calculating residual stresses.

## 4. Conclusions

The use of an open-source code for injection molding simulation with crystallization modeling was demonstrated. A validation was provided with the comparison of the predicted and the measured cavity pressure evolutions. While other research focused on iPP, the HDPE crystallization was inspected. The Kolmogorof-Avrami-Evans crystallization model was calibrated according to fast scanning calorimetry studies and insight into relevant cooling rates and solidification temperatures was gained. This can, for instance, serve as guidance in selecting the no-flow temperature or adjusting the two-domain Tait equation in industrial simulation.

The injection molding simulation research can be conducted using *OpenFOAM*, an open source CFD package, allowing the research to focus to the physics by utilizing the established CFD workflows. The amount of the required coding to develop similar solutions was thus greatly reduced.

The use of *OpenFOAM* has important implications for further research. Being a state-of-the-art general purpose CFD code while also open source it allows for advanced numerical modeling, with options for tailoring the numerical solution by altering the linear system of equations’ solvers and appropriate differencing schemes. Advanced modeling approaches of dynamic meshing allowed modeling a deformable cavity but could be used to model compression molding etc.

The utilized code and simulation setup are publicly available including the code for generating the graphical results. This allows the researchers to probe the influence of any of the listed parameters, including cavity thickness or flow-path length. The modeling can be further developed. The crystallization model can be updated to include flow induced crystallization and to predict non-spherulitic morphology. As for the general features, modeling of gates, venting and in-mold shrinkage would contribute a great deal to its industrial applicability. Ultimately, it could be computationally optimized to also serve the industry where accuracy is often sacrificed to reduce computational times.

## Figures and Tables

**Table 1:** Crystallization parameters.

Symbol	Value	Unit
$N_{0}$	1 × 10^8^	m^3^
$\Delta T_{0}$	2.5	K
$T_{m}^{0}$	419	K
$G_{k,0}$	103	m/s
$E_{D}$	29.3	kJ/mol
$C_{3}$	265	K
$r_{c}$	1.5	1

**Table 2:** Tait equation parameters for HDPE Sabic M80064 [35].

Symbol	Melt	Melt and Solid	Solid	Unit
$b_{1}$	$12.74\times 10^{-4}$	/	$10.75\times 10^{-4}$	$m^{3}/\mathrm{kg}$
$b_{2}$	$10.26\times 10^{-7}$	/	$2.077\times 10^{-7}$	$m^{3}/\left(\mathrm{kgK}\right)$
$b_{3}$	$9.263\times 10^{7}$	/	$33.24\times 10^{7}$	Pa
$b_{4}$	$4.941\times 10^{-3}$	/	$2.46\times 10^{-6}$	$K^{-1}$
$b_{5}$	/	414.5	/	K
$b_{6}$	/	$1.543\times 10^{-7}$	/	K/Pa
$b_{7}$	/	$1.872\times 10^{-3}$	/	$m^{3}/\mathrm{kg}$
$b_{8}$	/	$5.158\times 10^{-2}$	/	$K^{-1}$
$b_{9}$	/	$1.023\times 10^{-8}$	/	${\mathrm{Pa}}^{-1}$

**Table 3:** Cross-WLF viscosity model parameters (modified from [35]).

Symbol	Value	Unit
n	0.394	1
$\tau^{*}$	64.57	kPa
$D_{1}$	$3.76\times 10^{15}$	Pa/s
$D_{2}$	153.15	K
$D_{3}$	0.15	K/MPa
$A_{1}$	33.21	1
${\tilde{A}}_{2}$	51.6	K

**Table 4:** Parameters of the empirical viscosity modification due to crystallization.

Symbol	Value	Unit
f	1000	1
h	0.2	1
m	2	1

## Data Availability

The data presented in this study are openly available on Zenodo at https://doi.org/10.5281/zenodo.4314423.
